# Lung infection with classical *Klebsiella pneumoniae* strains establishes robust macrophage-dependent protection against heterologous reinfection

**DOI:** 10.1016/j.micinf.2024.105369

**Published:** 2024-05-28

**Authors:** Joseph J. Mackel, Casey L.G. Mick, Emily Guo, David A. Rosen

**Affiliations:** a Department of Pediatrics, Division of Infectious Diseases, Washington University School of Medicine, St. Louis, MO 63110, USA; b Department of Molecular Microbiology, Washington University School of Medicine, St. Louis, MO 63110, USA

**Keywords:** *Klebsiella pneumoniae*, Pneumonia, Heterologous infection, Lung immunity, Lung macrophage, Clodronate depletion

## Abstract

At present, there is no approved vaccine for prevention of infection by the opportunistic bacterium *Klebsiella pneumoniae* (Kp); success in treating these infections is increasingly challenged by the spread of antibiotic resistance. Preclinical investigation of adaptive immunity elicited by lung infection with live classical Kp may reveal host mechanisms of protection against this pathogen. Here, we utilize multiple virulent classical Kp strains to demonstrate that following lung infection, surviving wild-type mice develop protective immunity against both homologous and heterologous (heterotypic) reinfection. For Kp strains with low capacity to disseminate from the lung, this immunity is B-cell-independent. We further demonstrate that this immune protection is also effective against subsequent challenge with hypervirulent Kp if the strains share the same capsule type. Systemic inoculation fails to elicit the same protective effect as lung inoculation, revealing a lung-specific immune effector function is responsible for this protection. We therefore utilized clodronate-loaded liposomes to substantially deplete both alveolar macrophages and lung interstitial macrophages, finding that simultaneous depletion of both subsets entirely ablates protection. These findings indicate that following initial lung infection with Kp, lung macrophages mediate protection against ensuing Kp challenge.

## 1. Introduction

*Klebsiella pneumoniae* (Kp) is a Gram-negative bacterium that causes a variety of human infections. Traditionally, risk for Kp infection has been associated with immunocompromised states, as innate immune defenses are normally sufficient to clear classical Kp (cKp) strains in healthy individuals. In recent decades, hypervirulent Kp (hvKp) strains that can infect healthy hosts have emerged as a new threat to public health. Further, resistance to available antibiotic treatments is spreading among both classical and hypervirulent strains, creating a critical need for additional strategies to prevent and treat Kp infections [1]. *Klebsiella* species are a leading agent of hospital-acquired infection following SARS-CoV-2 infection, possibly due to high rate of mechanical ventilation [2]. The COVID-19 pandemic likely accelerated the spread of antibiotic-resistant Kp due to stress on healthcare systems and increased numbers of antibiotic-exposed, seriously ill patients [3].

Further insight into the pathogenesis of cKp strains and the host defense mechanisms required for protection may accelerate the development of novel interventions. Historically, preclinical studies in mice have focused primarily on the hvKp strain 43816; however, more recent mouse models have expanded the study of cKp strains [4]. Study of diverse strains has revealed differential immune effector requirements for control and clearance of different Kp strains during primary infection [5]. Using the cKp strain TOP52, we developed a model of low-dose lung inoculation followed 28 days later by high-dose rechallenge to dissect mechanisms of adaptive immunity and trained innate immunity. In this model, TOP52 lung infection elicits protective immunity against homologous rechallenge [6]. Here, we test the efficacy of this protection against heterologous challenge with both cKp and hvKp strains and investigate the effector responses associated with this protective phenotype.

## 2. Materials and methods

### 2.1. Bacterial preparations

Infections were performed with cKp strains TOP52 [6–10], KR3 [10], or KR174 [10], or with hvKp strain 43816 [11] (Table 1). Strains were sequenced and then analyzed with Pathogenwatch (https://pathogen.watch/) for determination of capsule and O-antigen types. Bacteria were grown statically in 20-mL cultures of Luria–Bertani (LB) broth overnight at 37°C. Cultures were centrifuged at 8000×*g* for 10 min, and bacteria were subsequently resuspended in sterile phosphate-buffered saline (PBS) and diluted to the desired inoculum concentration by measuring optical density at 600 nm (OD_600_). Inocula were verified by serial dilution and plating. For inoculations utilizing heat-killed bacteria, inocula were incubated at 60°C for 30 min, and lack of viability was confirmed via plating.

### 2.2. Mouse infections

All animal procedures complied with ethical regulations for animal testing and research and were approved by the Institutional Animal Care and Use Committee at Washington University School of Medicine. Mice aged 7–8 weeks at the initiation of each experiment were used for all studies. Wild-type mice C57BL/6J (000664) were acquired from The Jackson Laboratory and acclimated for one week prior to use in experiments. B-cell deficient mice (μMT^−/−^; B6.129S2-*Ighm*^*tm1Cgn*^/J; 002288) were also initially acquired from The Jackson Laboratory and bred in Washington University animal facilities. To avoid confounding of experimental results with variations in microbiome or other factors due to differences in animal facilities, experiments were designed to avoid direct comparisons of wild-type (WT) with *μMT*^−/−^ mice. Mice were infected using a non-surgical oropharyngeal aspiration method [10] in which mice were anesthetized with inhaled isoflurane and suspended by the upper incisors. The tongue was then retracted laterally and inoculum (50 μL volume) was administered via pipet to the oropharynx and aspirated on subsequent breath. For intraperitoneal infections, inoculum (100 μL) was delivered into the right lower quadrant of the abdomen utilizing a 29-gauge needle. For survival experiments, mice were monitored for morbidity and mortality and weighed daily for 14 days following challenge. For lung bacterial titers, lungs were collected into PBS, homogenized, serially diluted, and plated on LB agar to determine organ bacterial titers. For blood cultures, ~100–200 μL blood was collected via submandibular puncture immediately prior to sacrifice, into tubes containing 5 μL of sterile-filtered 0.5M EDTA. Blood was serially diluted and plated on LB agar.

### 2.3. Macrophage depletions

Macrophages were depleted with clodronate liposomes (Liposoma). Control mice were administered liposomes containing PBS. Liposomes were warmed to room temperature prior to administration. For depletion of alveolar macrophages, mice were administered 50 μL liposomes (5 mg/mL clodronate or control) via oropharyngeal aspiration. For depletion of interstitial macrophages, mice were administered 200 μL liposomes via retroorbital injection [12].

### 2.4. Flow cytometry

For flow cytometric analyses, lungs were resected, placed in sterile PBS, then transferred to 1 mL digestion media [2.5 mg/mL Collagenase D (Roche 11088866001) + 3% fetal bovine serum (FBS; Gibco 10438–026) in RPMI medium (Sigma R8758)], minced with scissors, and incubated for 1 h at 37°C with shaking at 200 rpm. Digested lung pieces were dissociated through 40-μm cell strainers and washed with FACS buffer [PBS + 1% FBS (Gibco 10438–026) + 0.9% sodium azide]. All centrifugation steps were performed in a swinging bucket centrifuge at 300×*g*. Red blood cells were lysed with Pharm Lyse Buffer (BD Biosciences 555899). Cells were washed, then resuspended in PBS and stained with LIVE/DEAD^™^ Fixable Blue Dead Cell Stain and blocked with Fc Block (BD Biosciences 553142) for 30 min at room temperature. Cells were washed, resuspended in FACS buffer, and stained at 4°C for 30 min with CD45-BV510 (Biolegend 103138), CD3-APCCy7 (Biolegend 100222), CD19-BUV661 (BD Horizon) Ly6G-Alexa700 (Biolegend 127621), CD64-PE (Biolegend 139304), SiglecF-Alexa647 (BD Pharmingen 562680), CD11c-BV785 (Biolegend 117336), and F4/80-BB700 (BD OptiBuild 746070). After staining, cells were washed then fixed in 2% paraformaldehyde until acquisition on a Cytek Aurora 5 Laser Cytometer. Single-color controls for compensation were prepared with Invitrogen UltraComp eBeads (01–2222-42), except for the viability dye, which was prepared with a mixture of heat-killed and viable cells. Total cell counts per organ were calculated using Precision Count Beads (Biolegend 424902) according to the manufacturer’s instructions.

### 2.5. Statistics

For Kaplan–Meier survival analyses, the Mantel–Cox log-rank test was used to determine differences in survival between two groups. Student’s *t*-test with Holm-Sidak correction for multiple comparisons was used to determine differences in mouse weights. The Mann–Whitney U-test was used for comparisons between cell counts obtained via flow cytometry and organ titers, as they were not all normally distributed. All tests were two-tailed, and P-values <0.05 were considered significant. Analyses were performed using GraphPad Prism 10.2.

## 3. Results

### 3.1. Pathogenicity of diverse clinical cKp isolates

Kp strains isolated from patients are extremely genetically diverse; the impact of these differences on infections of various niches and patient outcomes is not thoroughly defined. Therefore, inclusion of multiple strains in pathogenesis and immunology preclinical research may yield results more pertinent to human translation. Our previous studies of Kp lung infection have utilized the classical KL46:O3b (capsule type: O-antigen type) isolate TOP52 (herein cKp-KL46). Among classical strains, capsule type and degree of pathogenicity (based on presence of virulence factors) can vary greatly. Therefore, we also utilized lower respiratory classical isolates KR3 (KL22:O1; herein cKp-KL22) and KR174 (K2:O1; herein cKp-K2) to interrogate the similarities and differences in pathogenesis and immunity to clinically relevant strains (Table 1). These strains differ in capsule type, antibiotic resistance profiles, and likely numerous other aspects of their accessory genomes. HvKp strains often exhibit an LD_50_ < 10^2^ CFU in WT mice, while LD_50_ values of classical strains are generally ≥ 10^7^ CFU [13]. Although capsule type K2 is highly associated with hypervirulence, cKp-K2 is not a hypervirulent strain, as it lacks typical hypervirulence genes or hypervirulence plasmids and was isolated from a patient with a classical disease syndrome. Further, lung inoculation with 10^7^ CFU cKp-K2 resulted in no lethality in mice (data not shown).

**Fig. 1.**
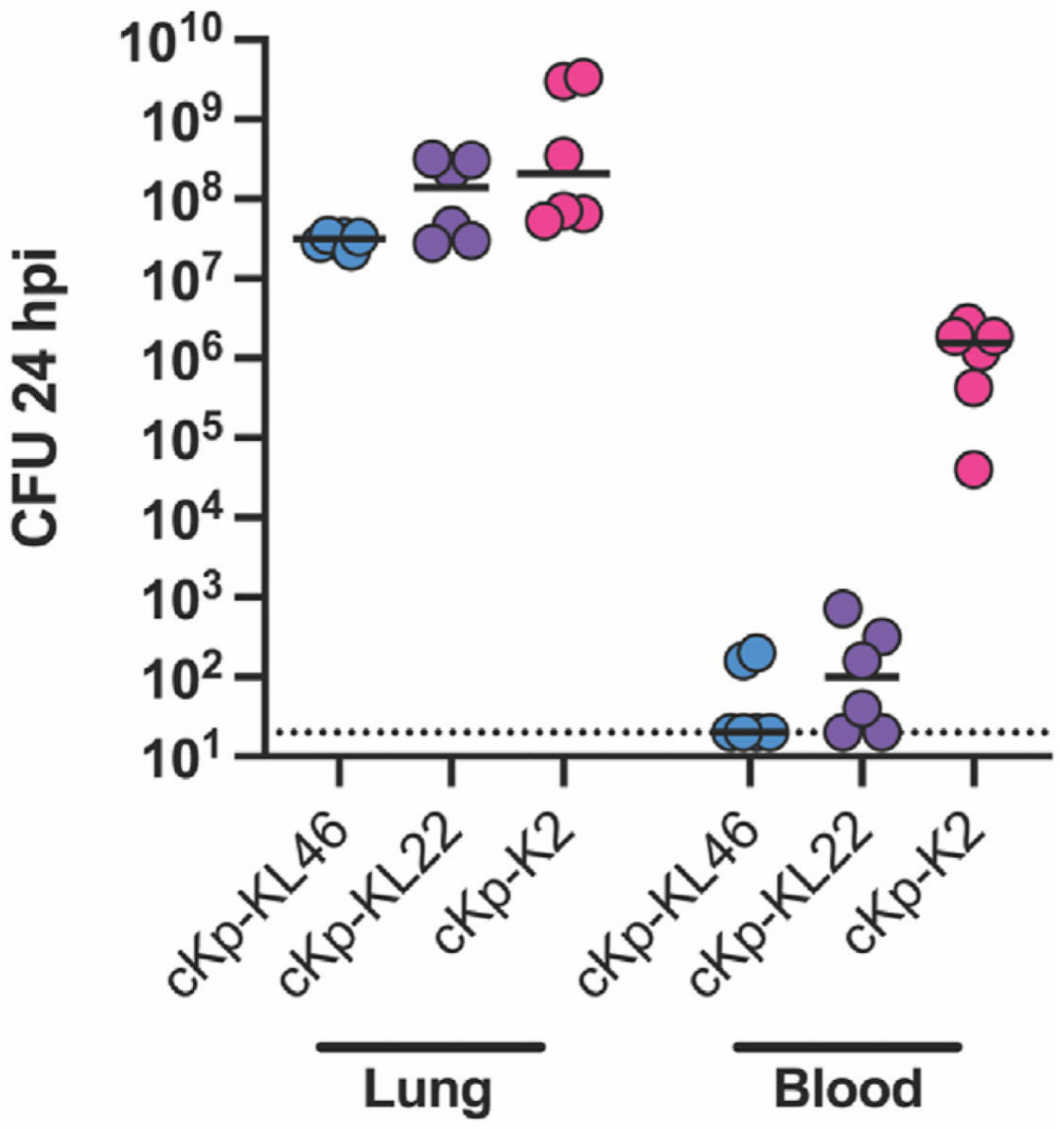
*Klebsiella pneumoniae* clinical isolates exhibit differences in pathogenicity in a mouse pneumonia model. Mice were infected via oropharyngeal aspiration with 10^8^ CFU live cKp-KL46, cKp-KL22, or cKp-K2. (A) Lung (CFU/lung) and blood (CFU/mL) titers 24 hpi. (n = 6 mice/group, 2 independent experiments) Horizontal bar indicates median value for each group. The dotted line indicates assay limit of detection.

We infected naïve WT C57BL/6J mice with 10^8^ CFU cKp (cKp-KL46, cKp-KL22, or cKp-K2) via oropharyngeal aspiration and assessed lung and blood titers 24 hours post infection (hpi) (Fig. 1). All three isolates produced high lung burden 24 hpi. Strikingly, cKp-KL46 and cKp-KL22 resulted in negligible dissemination into the blood, while cKp-K2 reached 10^6^ CFU/mL in the blood. We have classified cKp-K2 as a “high disseminator” in our model, while cKp-KL22 is a “low disseminator”. Thus, classical clinical isolates vary in both degree of pathogenicity and propensity to disseminate following lung infection in our pneumonia model.

### 3.2. B-cell-independent protection against reinfection

**Fig. 2.**
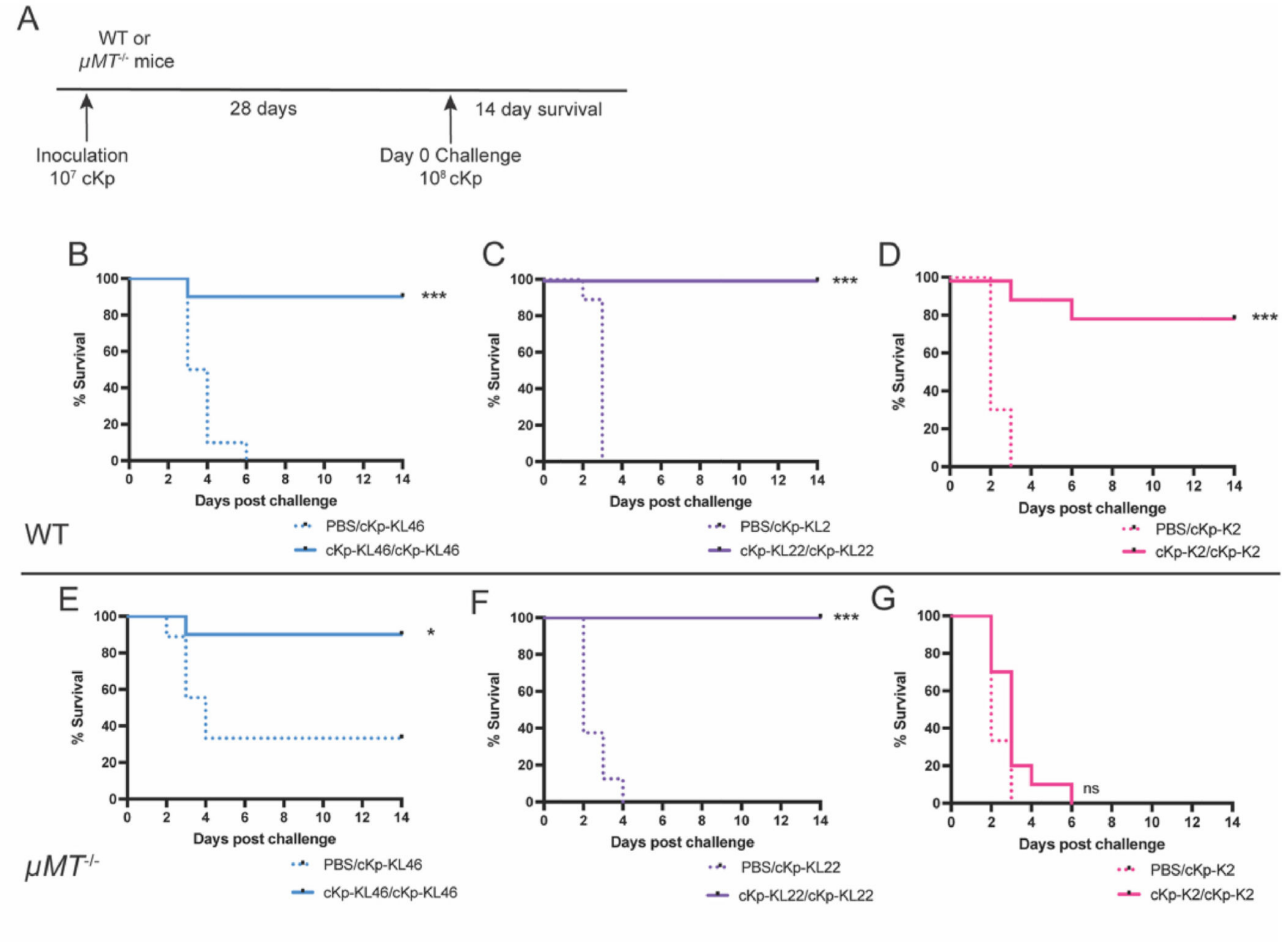
Lung inoculation with live cKp establishes protection against homologous challenge that is not dependent on B cells. (A) Scheme indicating mice were inoculated with 10^7^ CFU live cKp via oropharyngeal aspiration and challenged 28 days later with 10^8^ CFU of the same strain. Survival following challenge in WT mice (B–D) and μMT^−/−^ mice (E–G) (n = 8–10 mice/group, 2–3 independent experiments/group). *** indicates p < 0.001. * indicates p < 0.05. ns indicates not significant.

We have previously reported that lung inoculation with live cKp-KL46 generates protective immunity against high-dose challenge with the same strain [14]. Following inoculation, mice return to baseline weight within approximately 7 days (Fig. S1) and the inoculation dose is cleared from the lung prior to challenge [14]. We first set out to confirm that this protection is generated in response to multiple classical strains in addition to cKp-KL46. We inoculated mice with 10^7^ CFU of live cKp-KL46, cKp-KL22, or cKp-K2 and 28 days later challenged each group with 10^8^ CFU of the same strain with which they were initially inoculated (Fig. 2A). In each case, mice were protected against subsequent homologous challenge (Fig. 2B–D).

To begin dissecting the immune effectors mediating this protection, we first focused on antibody responses, the correlate of immune protection most often associated with bacterial vaccines. We employed μMT^−/−^ mice to determine if antibodies are required for protection in our repeat infection model. These mice lack mature B cells and thus lack the ability to produce functional antibodies. μMT^−/−^ mice underwent the lung inoculation plus challenge as described above for WT mice (Fig. 2A). Strikingly, B cells were not required for protection against homologous challenge with the low-disseminating strains cKp-KL46 (Fig. 2E) or cKp-KL22 (Fig. 2F), as demonstrated by survival of previously exposed μMT^−/−^ mice following challenge. However, in the case of the high disseminator cKp-K2, μMT^−/−^ mice did not gain a protective benefit from initial inoculation (Fig. 2G), indicating that antibody responses are critical for protection against challenge with this high-disseminating cKp strain. These experiments demonstrate that in this pneumonia model antibody responses are dispensable against these low-disseminating strains and required for a high-disseminating strain.

### 3.3. Heterologous reinfections with cKp

The genetic diversity present within the Kp microbial population, particularly among capsule types, requires development of prophylactic strategies with efficacy against diverse clinical strains. As our data in Fig. 2 suggested an antibody-independent effector response is protective against multiple cKp strains, we further investigated this response as an alternative or complementary strategy for the generation of broadly effective protection. We hypothesized that this alternative response might be less specific and instead broadly protective against challenge with Kp strains beyond the inoculation strain.

**Fig. 3.**
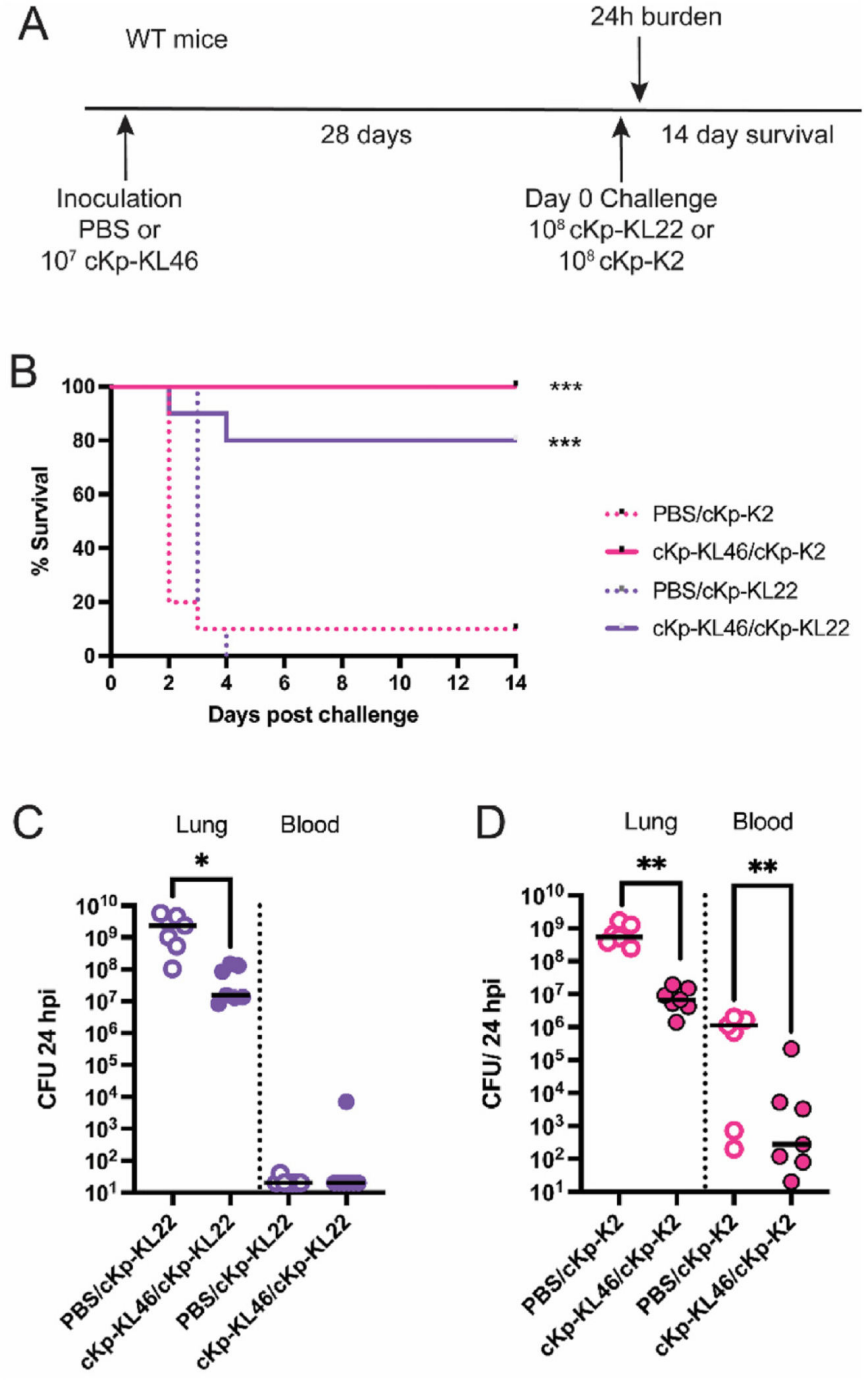
cKp inoculation provides cross-protection against heterologous challenge. (A) Scheme indicating WT mice were inoculated with 10^7^ CFU live cKp-KL46 via oropharyngeal aspiration and 28 days later challenged with 10^8^ CFU of cKp-K2 or cKp-KL22. (B) Survival following challenge, lung and blood burden 24 hpi with (C) cKp-KL22 or (D) cKp-K2 (n = 10 mice/group for survival studies and n = 8 mice/group for burden studies, 2 independent experiments). *** indicates p < 0.001. ** indicates p < 0.01. * indicates p < 0.05.

To test this hypothesis, we performed heterologous infection experiments in which mice were inoculated with cKp-KL46 and then challenged with either cKp-KL22 or cKp-K2 (Fig. 3A). We observed significant protection as measured by survival (Fig. 3B) and bacterial burden (Fig. 3C and D) during heterologous challenge infections compared to mock-inoculated controls, indicating that initial inoculation with live classical Kp establishes an immune response that is protective against respiratory tract challenge with diverse strains. Lung burden was decreased in both heterologous infections, and blood titers were significantly reduced during challenge with the high-disseminating cKp-K2 (Fig. 3C and D). These experiments demonstrate that lung inoculation with cKp establishes robust protection against challenge with heterologous high- or low-disseminating cKp strains.

### 3.4. Heterologous reinfections with hvKp

**Fig. 4.**
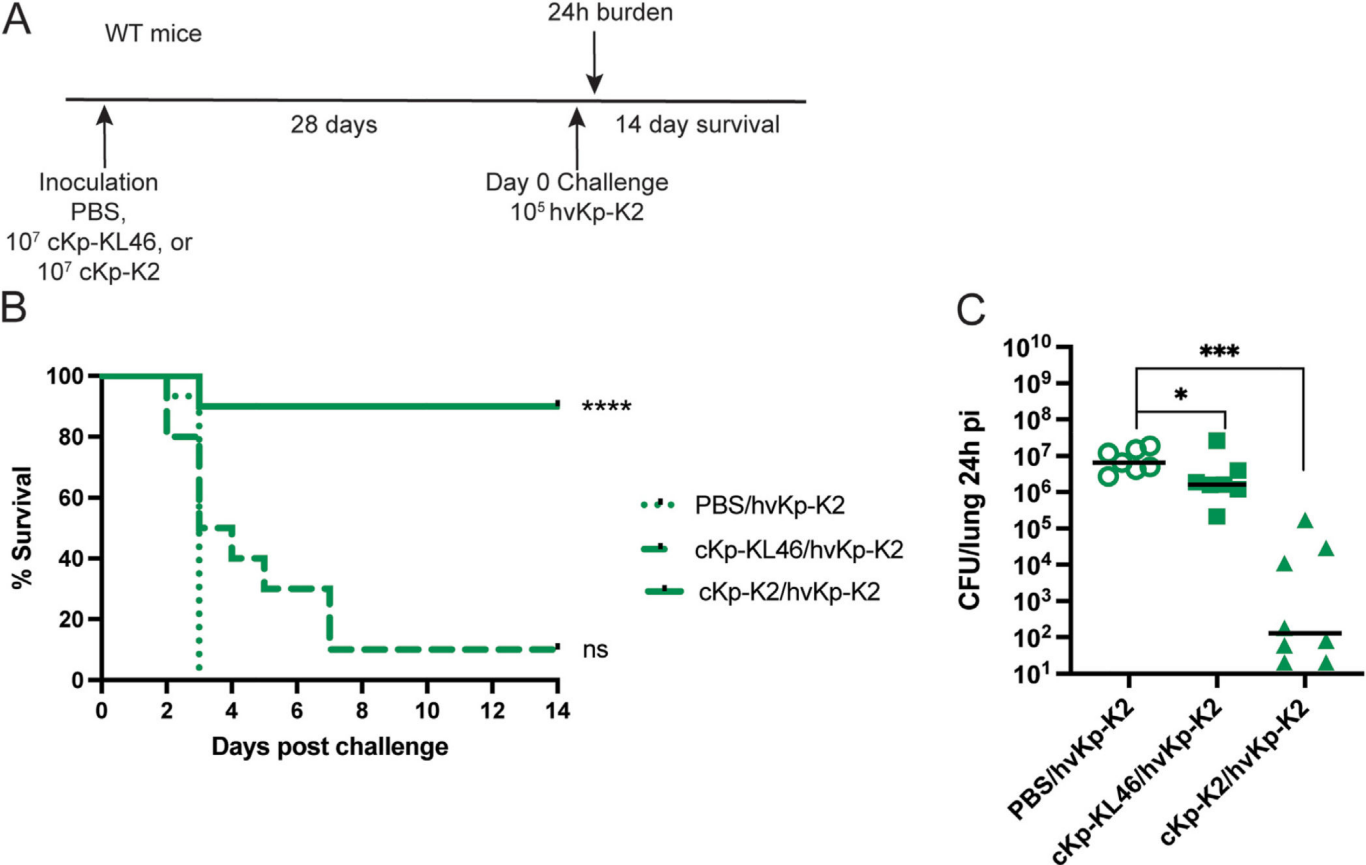
Cross-protection against heterologous challenge with hvKp requires matched capsule type. (A) Scheme indicating WT mice were inoculated with 10^7^ CFU live cKp-KL46 or cKp-K2 via oropharyngeal aspiration and 28 days later challenged with 10^5^ CFU hvKp-K2. (B) Survival following challenge and (C) lung burden 24 hpi following hvKp-K2 challenge (n = 10–15 mice/group for survival studies and n = 8 mice/group for burden studies, 2 independent experiments). *** indicates p < 0.001. ** indicates p < 0.01. * indicates p < 0.05.

Next, we asked if the protective response generated by inoculation with cKp is sufficient to protect against challenge with a hypervirulent strain (Fig. 4A). We employed the widely studied hypervirulent strain 43816 (herein hvKp-K2), which bears the same capsular type as cKp-K2. We observed that inoculation with cKp-KL46 provided no protection against mortality from hvKp-K2 challenge (Fig. 4B) and only modest improvement in bacterial burden (Fig. 4C). However, lung inoculation with cKp-K2 established robust protection against hvKp-K2 infection (Fig. 4B) and significant improvement in bacterial burden (Fig. 4C). Thus, lung cKp inoculation elicits protection against serotype-matched hvKp challenge, likely reflecting activity of anti-K2 capsule antibodies.

### 3.5. Lung versus systemic inoculation

**Fig. 5.**
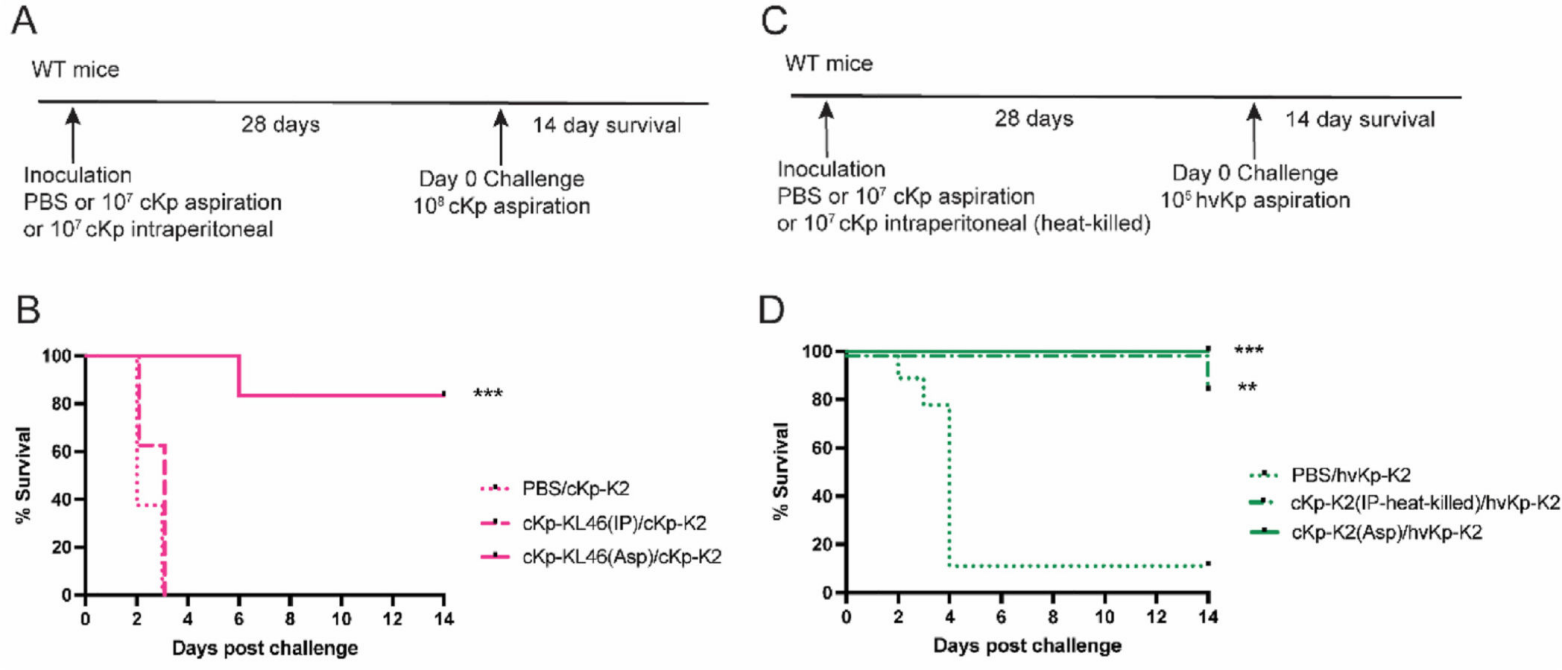
Systemic inoculation is not sufficient for lung protection against cKp challenge. (A) Scheme indicating WT mice were inoculated with 10^7^ CFU live cKp-KL46 via oropharyngeal aspiration or intraperitoneal injection and 28 days later challenged with 10^8^ CFU cKp-K2. (B) Survival following challenge of the groups described in A. (C) Scheme indicating WT mice were inoculated with 10^7^ CFU live cKp-K2 via oropharyngeal aspiration or 10^7^ CFU heat-killed cKp-K2 via intraperitoneal injection and 28 days later challenged with 10^5^ CFU hvKp-K2. (B) Survival following challenge of the groups described in C. (n = 10–15 mice/group for survival studies and n = 8 mice/group for burden studies, 2 independent experiments). *** indicates p < 0.001. ** indicates p < 0.01.

We have previously reported, after lung inoculation with cKp-KL46, that circulating T cells are not required for protection from homologous challenge [14]. We have now also excluded antibody responses as requisite effectors for protection in our model (Fig. 2). These data suggest that a tissue-specific response in the lung is responsible for protection against low-disseminating cKp strains. To test this hypothesis, we inoculated mice with cKp-KL46, either in the lung (to generate local and systemic responses) or in the peritoneum (to generate only systemic responses) and subsequently challenged the mice with cKp-K2 lung infection (Fig. 5A). Intraperitoneal inoculation provided no protection against lung challenge, while mice originally inoculated in the lung were protected from challenge, confirming a lung-specific protective response (Fig. 5B).

We performed a similar experiment with cKp-K2 lung or peritoneal inoculation followed by hvKp-K2 challenge in the lung. These inoculations were performed with heat-killed cKp-K2 because this strain exhibits increased virulence in peritoneal versus lung inoculation. In the case of this hvKp strain, either inoculation (in the lung or the peritoneum) was sufficient for protection against challenge with hvKp-K2 (Fig. 5C). These results support the critical importance of systemic anti-capsule antibodies in control of hypervirulent infections and implicate additional, lung-specific effector functions for control of lung infection with cKp strains.

### 3.6. Functional requirement for lung macrophages in protection against challenge

**Fig. 6.**
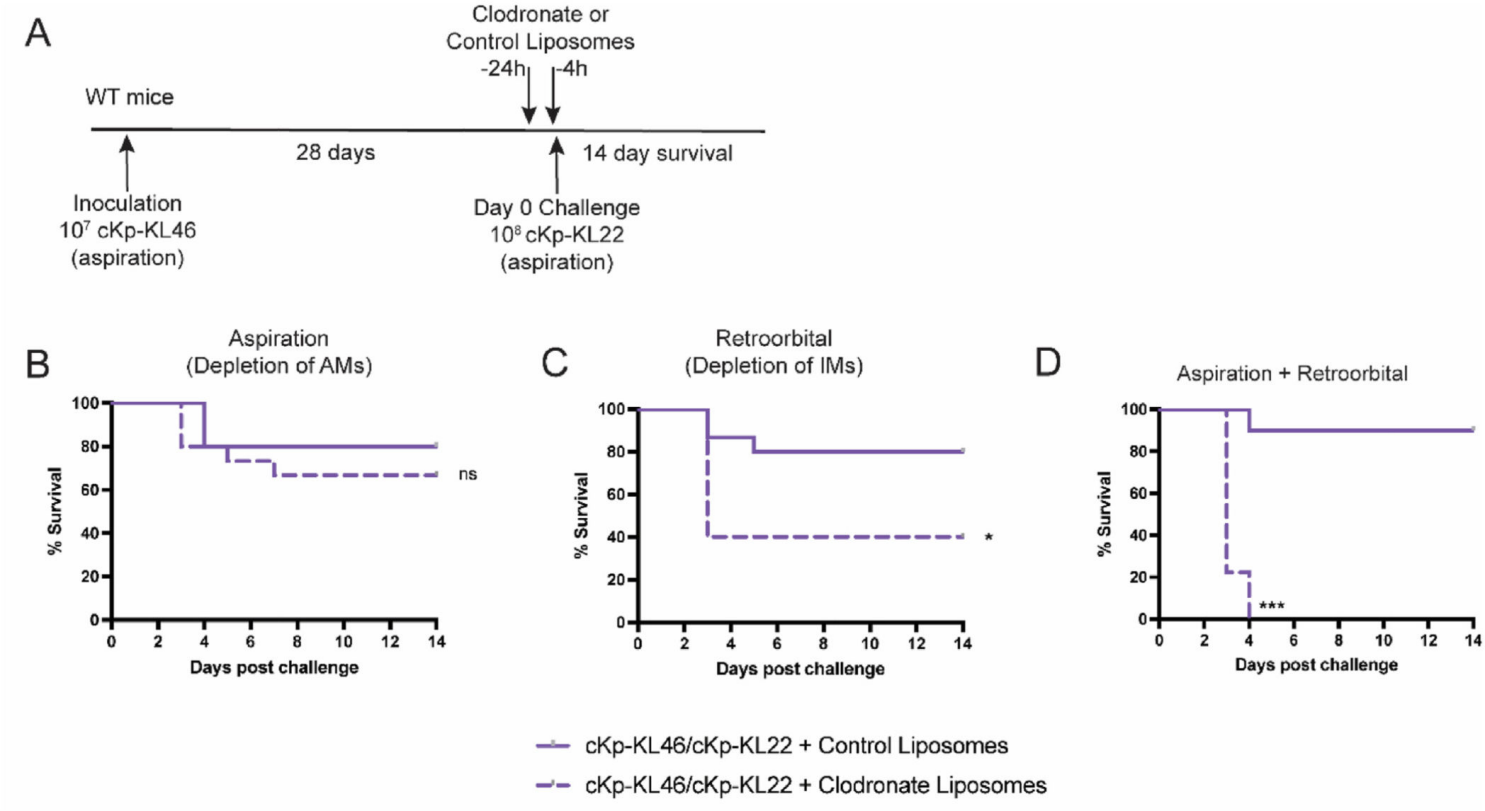
Lung macrophage depletion diminishes protection against heterologous challenge. (A) Scheme indicating WT, previously inoculated mice were administered clodronate (or control) liposomes for depletion of alveolar and/or lung interstitial macrophages prior to challenge with 10^8^ CFU cKp-KL22. Survival following challenge of mice partially depleted of (B) alveolar macrophages (C) interstitial macrophages, or (D) both alveolar and interstitial macrophages. (n = 9–15 mice/group, 2–3 independent experiments). *** indicates p < 0.001. * indicates p < 0.05.

We hypothesized that the lung-specific effector might be a macrophage population that is expanded, recruited, or activated following inoculation. To determine if lung macrophages were functionally important for protection, we administered clodronate-loaded or control liposomes to previously inoculated mice via oropharyngeal aspiration (for depletion of alveolar macrophages [AMs]) or via retroorbital injection (for depletion of interstitial macrophages [IMs]) [12]. In an additional group of mice, we depleted both AMs and IMs. We subsequently performed heterologous challenge in these groups of mice and assessed survival (Fig. 6A). Subsets of each group of control and depletion mice were sacrificed for analysis of depletion efficiency. As expected, aspiration of liposomes significantly depleted AMs but did not affect lung IM numbers. Retroorbital administration of liposomes significantly depleted lung IMs but did not affect AMs. Dual administration of liposomes by both routes successfully decreased numbers of both subsets (Figure S2).

In our reinfection model, following cKp inoculation, we observed that depletion of AMs alone did not affect susceptibility to cKp challenge (Fig. 6B). In contrast, mice partially depleted of IMs alone (Fig. 6C) or both AMs and IMs simultaneously (Fig. 6D) were significantly more susceptible to cKp challenge compared to control mice. These data indicate that IMs are required for the protective phenotype. The additive effect of simultaneous depletion of both subsets suggests that AMs also contribute to protection but are dispensable in the presence of IMs.

## 4. Discussion

Due to surging drug resistance, once-treatable infections caused by Kp and related Gram-negative bacteria are now gravely concerning. Various vaccination strategies have been proposed but are yet to be fully developed and approved. Historically, preclinical models of adaptive immunity to Kp have focused on hypervirulent Kp, but recently murine models of cKp pneumonia have been employed to begin to understand the host response to cKp lung infection. Our model employs a relatively low inoculation dose, which causes mild disease in immunocompetent mice to mimic a theoretical natural infection in humans. While immunocompetent humans are not at risk of developing cKp pneumonia, we contend that this model infection in an immunocompetent host can be used to dissect adaptive or trained innate immune mechanisms that render healthy hosts impervious to cKp infection at a much higher challenge dose. These mechanisms could then potentially be modulated in immunocompromised hosts to gain protection against infection. In the current work, we report that primary cKp lung infection establishes a protective response against reinfection, which is dependent on lung macrophages and not antibodies. In the case of high-disseminating strains, a specific antibody response is required. The role of macrophages in protection against high-disseminating Kp is not directly explored in this work. The primary implication of these results is the identification of lung macrophages previously exposed to Kp as exceptional anti-Kp effectors during secondary exposure. Further investigation of the precise location and phenotypes of these macrophages for assessment of the potential of engaging these cells in prophylactic or therapeutic strategies is warranted.

Following primary lung infection, a host may be rendered more susceptible to secondary infection or may develop protective immunity resulting in resistance to subsequent infections [15]. In a two-hit mouse model, mice subjected to *Escherichia coli* pneumonia were protected from subsequent *Pseudomonas aeruginosa* lung challenge [16]. Conversely, another model demonstrated increased bacterial burden and alveolar macrophage paralysis during secondary pneumonia following *E. coli* or *Staphylococcus aureus* primary pneumonia [17]. Mild pneumococcal infection results in immunity to subsequent heterologous challenge [18]. Regarding Kp, we have previously reported that primary Kp pneumonia is protective against subsequent homologous challenge [6,14]. We further found that T cell responses are not required as immune effectors during homologous cKp challenge [14]. Here, we set out to dissect the contributions of potentially protective antibody and lung macrophage responses during homologous and heterologous cKp challenge. For these studies, we employed cKp clinical isolates that produce high lung burden in WT (immunocompetent) mice (Fig. 1). Of these isolates, cKp-KL46 and cKp-KL22 have low ability to disseminate from the lung and survive in the blood, modeling pneumonia in the absence of dissemination to other organs. The third isolate, cKp-K2 causes high lung burden and disseminates in the blood. Utilizing these diverse isolates enables analysis of immune responses effective against the breadth of cKp pathogenic modalities.

Generation of antibody responses through vaccination is a fundamental approach for prevention of bacterial infection. We have previously demonstrated that passive administration of circulating antibodies does not lessen severity of high-dose cKp lung challenge [14] and here report the ability of B-cell deficient mice inoculated with cKp-KL46 or cKp-KL22 to survive a high-dose homologous challenge similarly to WT mice (Fig. 2). These results demonstrate that antibody responses are dispensable for protection against two low-disseminating classical strains. Strikingly, this is not the case for protection against cKp-K2, which is not achieved in the absence of B cells and associated antibodies. K2 capsule is known to evade killing in the blood independent of other characteristics of hypervirulence [19], which corresponds with our data that cKp-K2 effectively disseminates from the lung and survives in the blood (Fig. 1). As demonstrated in Fig. 3, inoculation with a non-capsule-matched strain generates protection, thus an antibody response to a non-capsular antigen must be sufficient for protection. Although the particular antigen remains elusive, it is clear that some antibody response is required for protection against this strain. We demonstrate that systemic inoculation, which likely produces protection only via circulating antibodies, is protective against hvKp, but not cKp lung challenge (Fig. 5). These results further suggest that protection against cKp pneumonia is best achieved with effectors other than antibodies, while protection against hvKp strains can be achieved primarily through antibody. Importantly, our results do not preclude that vaccination strategies based solely on antibody responses could be effective against low-disseminating classical strains, but suggest they are not the primary means by which the host establishes protection in this model. Thus, other potentially complementary effectors should be considered in vaccination strategies.

We hypothesized that macrophages could be the lung effector population that contributes to protection against heterologous challenge. The diversity of lung macrophage subsets and their roles in homeostasis and disease is a topic of extensive research [20]. The two major populations of resident macrophages in lung are alveolar macrophages and interstitial macrophages. During primary infection in naïve animals, alveolar macrophages play a critical host defense role. During infection, lung macrophages may die and can be replaced via self-renewal or by expansion of recruited macrophages, which are shaped by the lung niche and take on characteristics of alveolar or interstitial macrophages [21]. The inflammatory environment during and following infection may also impact the activation state and characteristics of macrophages [20]. We depleted either alveolar macrophages, interstitial macrophages, or both via clodronate liposomes and found that the protective effect of inoculation is lost entirely following simultaneous depletion of both alveolar and interstitial macrophages. Protection is partially lost following depletion of interstitial macrophages alone, suggesting that these cells are important for protection during challenge (Fig. 6). A central role for IMs in cKp control during secondary infection is supported by an earlier study, which found that IMs are the primary population of macrophages associated with Kp during primary infection [12]. Future studies will employ additional methods for depletion of macrophages, as clodronate depletion is not complete or entirely specific. Outstanding questions that are the subject of ongoing studies are the activation state of macrophages following cKp inoculation and the durability of macrophage-dependent protection in our model. Ultimately, characterization of the precise phenotype of protective macrophages will enable precise targeting of these cells for prevention or treatment of infection in context of invasive surgeries or chemotherapeutic treatments. The bacterial factors to which the host responds to establish these macrophages is also under investigation via inoculations with cKp genetically depleted of virulence factors.

Mucosal vaccines directed against Kp have focused on the establishment of tissue-resident T cells [22–24]. These studies clearly demonstrate that T cells can be effective mediators of protection. Efficacy of antibacterial antibodies is also well established and we have recently demonstrated protection against Kp challenge with multiple vaccines against capsule and O-antigen [13,25]. Here, we add to this list macrophages, possibly a lung interstitial population, as auspicious targets for activation for protection against Kp pneumonia. Our inoculation plus challenge model utilizing live cKp mimics a human natural infection. In this model, the macrophage response is the predominant method of host immunity against reinfection, as evidenced by the loss of protection following depletion of macrophages but not in the absence of B (Fig. 2) or T cells [14]. There are important limitations of this work to be considered. The protective effects observed were assessed relatively soon (4 weeks) following inoculation. Considering the macrophage phenotypes reported, innate immune priming or training is likely the dominant effect in these experiments. Further work is needed to assess durability of the protection and to determine if other non-Kp inflammatory insults to the lung also induce this protective phenotype.

## 5. Conclusions

The data presented here demonstrate that lung exposure to a dose of cKp that causes only a mild infection results in subsequent protection against reinfection with heterologous strains. In the case of cKp strains that do not readily disseminate from the lung to the blood and other organs, an antibody response is dispensable for survival. In contrast, survival of infection with readily disseminating cKp strains or hvKp strains requires an antibody response. Depletion experiments emphasize a protective role for lung macrophages, particularly interstitial macrophages, in this protection. Thus, targeted modification of macrophage responses may be a promising approach to protect the lung from infection with diverse strains of Kp.

## Supplementary Material

Supplementary Material

## Figures and Tables

**Table 1:** *Klebsiella pneumoniae* strains.

Name	Other name	Capsule	O-antigen	Dissemination	Reference

cKp-KL46	TOP52	KL46	O3b	Low	[6–10]
cKp-KL22	KR3	KL22	O1	Low	[10]
cKp-K2	KR174	K2	O1	High	[10]
hvKp-K2	43816	K2	O1	High	[11]
