# Supplementary material for: Lung infection with classical Klebsiella pneumoniae strains establishes robust macrophage-dependent protection against heterologous reinfection
Source: Microbes Infect. Author manuscript; Available in PMC 2024 Dec 2. (PMC11602523; doi:10.1016/j.micinf.2024.105369)
Supplement: Supplementary Material [file NIHMS2003378-supplement-Supplementary_Material.docx]

Supplementary Figures


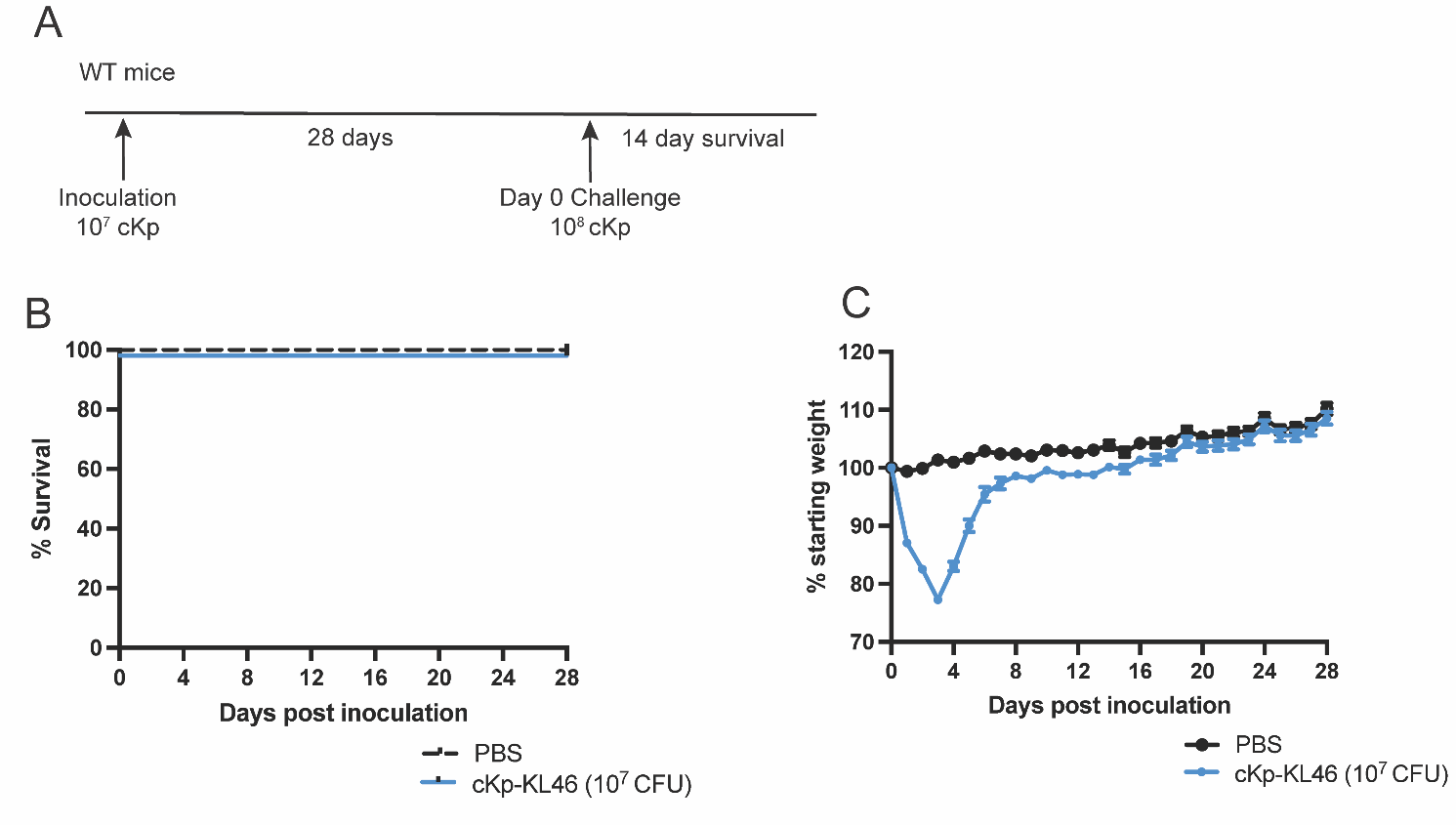


**Figure S1. Survival and weight loss of WT mice following inoculation.** (A) Scheme indicating WT mice were inoculated with 10^7^ CFU cKp-KL46 and subsequently challenged with 10^8^ CFU cKp-KL46. Survival (B) and weight change (C) following inoculation for 28 days prior to challenge (n=19-20) mice/group, 2 independent experiments).

**
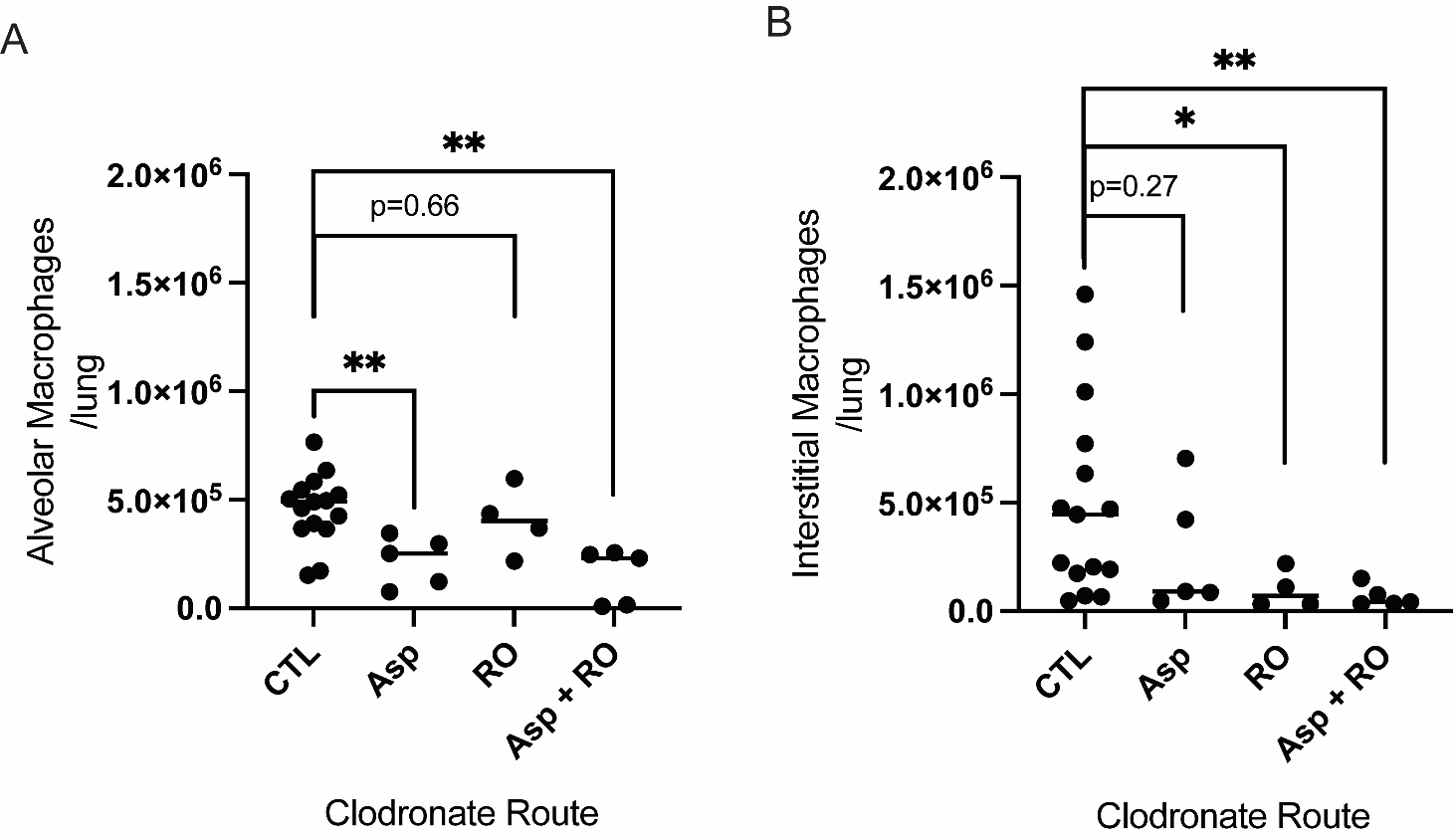
**

**Figure S2. Macrophage depletion following clodronate administration.** WT previously inoculated mice were administered clodronate (or control) liposomes either by aspiration (Asp) or retroorbital (RO) for depletion of alveolar and/or lung interstitial macrophages prior to challenge with 10^8^ CFU cKp-KL-22. Depletion efficiency was analysed via flow cytometry. Cells were gated on LIVE/DEAD-CD45+Ly6G-CD3-CD19- and then CD64-SiglecF+ cells were excluded. Within the remaining cells, lung macrophages were then identified as (A) F4/80+CD11c+ alveolar macrophages or (B) F4/80+CD11c- interstitial macrophages. (n=4-15 mice/group, 2 independent experiments). ** indicates p<0.01. * indicates p<0.05.
